# Immobilization of nematodes for live imaging using an agarose pad produced with a Vinyl Record

**DOI:** 10.17912/QG0J-VT85

**Published:** 2018-08-09

**Authors:** Katherine A Rivera Gomez, Mara Schvarzstein

**Affiliations:** 1 City University of New York (CUNY), Brooklyn College, Brooklyn, NY USA. x; 2 The Graduate Center at CUNY, NY, NY USA

**Figure 1. Overview of the process to make agar pads for live imaging of C. elegans using a vinyl record surface mold.**
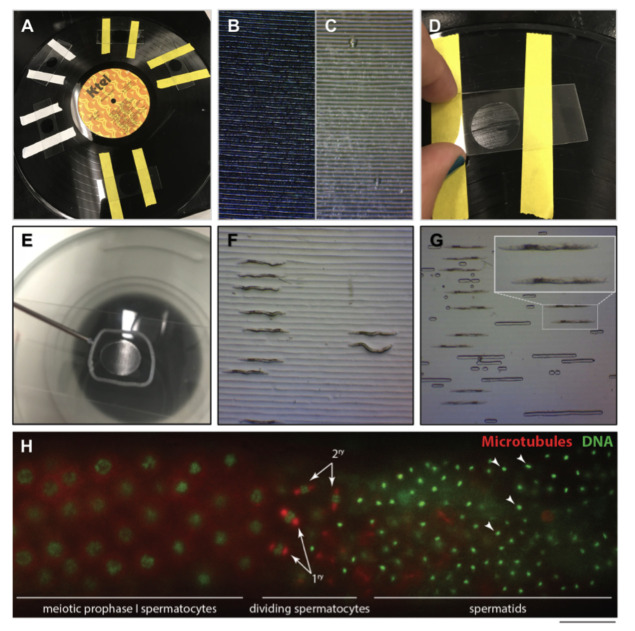
A. Laboratory label tape placed on a vinyl disc gives reference of where to place the agar drop and establishes an even pad thickness. B. Microgrooves on vinyl record. Space between microgrooves is approximately 80 µm wide. C. Agarose pad with grooves from vinyl record impression. The generated microgrooves are about 70-80 µm wide. D. Once agar solidifies slide is lifted with the attached agar pad. E. Petroleum jelly perimeter is applied around the agar pad. F. Anesthetized *C. elegans* males aligned in grooves before cover slip application. G. Males after slip cover application, in straight conformation. Inset shows two males lined up for live imaging of fluorescent gonads. H. Example of fluorescence microscopy live image using the grooved pads. Shown is the meiotic germline of a male mounted expressing mCherry::H2B histone and GFP::b-Tubulin, here false colored in green and red respectively for optimal contrast. Arrows show dividing primary and secondary spermatocytes; arrowheads show examples of spermatid nuclei. Scale bar is 15 µm.

## Description

Numerous microfluidic systems have been developed and used for live imaging of *Caenorhabditis* nematodes (Allen et al., 2008; Zhang et al., 2008; Krajniak and Lu, 2010; Krajniak et al., 2013; Cornaglia et al., 2015). These systems can be costly, complex to set up, or require high-maintenance between uses. In addition, microfluidic rigs can be thick, preventing live imaging of worms from strains expressing low fluorescence fusion proteins. In the absence of elaborate microfluidic rigs, most live imaging protocols utilize flat agarose pads along with anesthetics and/or microbeads to immobilize the nematodes (Kim et al., 2013). Since this method does not allow the user to maintain the nematode straight and does not prevent small movements that disturb live imaging, a higher number of worms need to be mounted to ensure that a some settle in an optimal position. This is especially problematic when trying to image nematodes genotypes that are scarce, since there is a very small number of nematodes with the desired genotype in a plate making it challenging to find enough animals to image. Here is a protocol, modified from Zhang, M. et al., 2008, to make grooved agarose pads utilizing a 12-inch vinyl Long Play (LP) record as a mold for agar pads in which nematodes can be positioned and immobilized for live imaging. This method is simple, effective, and allows long-term time-lapse imaging of young adult and adult hermaphrodites, and males expressing low fluorescence fusion proteins.

**Detailed protocol**First, place strips of labeling tape about 3 cm apart perpendicular to the grooves on a 12-inch diameter (331/3 rpm) vinyl LP’s grooved surface (Figure 1A,B). The distance between grooves on the LP is about 75-80 µm and when it is used as a mold grooves are created in an agarose pad similar to the width of young adults (Figure 1C). Place a drop of melted 4% agarose onto the vinyl record between the strips of tape. Immediately position the microscope slide on top of the drop to make a pad against the vinyl record. Once the agarose is solid, remove the microscope slide from the vinyl record by lifting from one side (Figure 1D). If an agar pad is too large, it can be trimmed down by using another microscope slide’s short flat edge to make straight cuts. Use a syringe filled with petroleum jelly and fitted with an 18-gauge blunt fill needle to make a line around the pad (Figure 1E). We have noticed that Vaseline**®** petroleum jelly is toxic to the animals when it touches the agarose pad. To ensure the agarose pad and the petroleum jelly don’t come into contact, the petroleum jelly line must be at least 2-3 mm away from the agarose pad. The petroleum jelly must form a closed circle to trap air and push the petroleum jelly away from the pad when the coverslip is added. VALAP may be used as an alternative sealant. Add volumes of 2-5 ml of anesthetic to the grooved pad (we use either levamisole [1mM] or serotonin [25mM-100mM] (Rog and Dernburg, 2015)). The anesthetic volume and concentration needed is dependent on the size of the pad and animal anesthetic resistance. Add the nematodes to the anesthetic on the agarose pad. When the worm movements have slowed down and most of the anesthetic has been absorbed into the pad, move the worms into the grooves utilizing a minutien pin or worm pick (Figure 1F). Worms are easier to position when most of the anesthetic has been absorbed into the pad, but do not allow agarose pad to dry completely as this will result in excessive air bubbles forming as the cover slip is put in place. Slightly press the coverslip over petroleum jelly ring evenly until it touches the pad and the worms are wedged tightly into the grooves (Figure 1G). As the coverslip is pressed down, the petroleum jelly circle will expand and make a seal around the pad to prevent evaporation of the anesthetics solution and desiccation of the nematodes. We found that using microbeads in conjunction with this protocol does not improve the immobilization of the nematodes. This method allows the user to quickly mount L4 or adult hermaphrodites, adult males, and keep them alive and in a straight position for at least 1 hour during time-lapse imaging of strains expressing low fluorescing fusion proteins (see example in Figure 1H).

## Reagents

12-inch long-playing (LP) vinyl record (331/3 rpm), labeling tape, Pasteur pipette with bulb, agar (at the concentration in which you prefer to use, we use 4% agar), microscope slides, your preferred anesthetic (we use either levamisole [1mM] or freshly made serotonin [25 mM-100 mM]), a minutien pin (or eyelash pick), coverslips, slide breathable sealant such as petroleum jelly filled syringe with an 18-gauge blunt fill needle or VALAP (1:1:1 by weight mixture of Vaseline:Lanolin:Paraffin).
